# PARTICIPANTS’ PERSPECTIVES ON RECEIVING RESULTS USING FOUND POETRY: A MIXED-METHODS STUDY

**DOI:** 10.1093/geroni/igad104.0256

**Published:** 2023-12-21

**Authors:** Sara Bybee, Jacqueline Eaton, Bob Wong

**Affiliations:** University of Utah, Salt Lake City, Utah, United States; University of Utah, Salt Lake City, Utah, United States; University of Utah, Salt Lake City, Utah, United States

## Abstract

Few researchers share study results with participants, often citing a lack of knowledge regarding how to do so. This study explored the dissemination preferences of study participants receiving results in the form of found poetry, developed from dyadic interviews with sexual and gender minority (SGM) and non-SGM couples’ regarding their experiences with cancer. Participants (N=24) completed demographic and health questionnaires, a pre-posttraumatic growth-inventory-expanded (PTGI-X), and were randomized to receive the found poem as text, text and audio, audio, or video formats. Participants completed a post-PTGI-X, reported dissemination preferences, and emotions experienced. Open-ended responses demonstrated that using found poetry to disseminate results communicated that participant experiences were understood, fostered introspection, and renewed appreciation for their partner. Only SGM participants (n=3) reported preferring a format other than the one to which they were randomized. The text-only and combined text and audio formats evoked the greatest number of emotions (n=13 each) followed by video (n=4), and audio-only (n=3). Across randomization groups, interest (n=11) was most frequently reported, followed by sadness (n=8), and joy (n=6). Quantitatively, there were no significant changes in participants’ PTG—a small sample size may have reduced statistical power—which might be seen as a failure. However, the open-ended responses indicate that innovative approaches to dissemination are acceptable and multiple options may increase accessibility. Only SGM participants preferred an alternate format, potentially reflecting a natural inclination to question the status quo. Including minority perspectives (positive/negative) illuminates the need to offer study participants choices in how and when they receive findings.

